# Stone heterogeneity index as the standard deviation of Hounsfield units: A novel predictor for shock-wave lithotripsy outcomes in ureter calculi

**DOI:** 10.1038/srep23988

**Published:** 2016-04-01

**Authors:** Joo Yong Lee, Jae Heon Kim, Dong Hyuk Kang, Doo Yong Chung, Dae Hun Lee, Hae Do Jung, Jong Kyou Kwon, Kang Su Cho

**Affiliations:** 1 Department of Urology, Severance Hospital, Urological Science Institute, Yonsei University College of Medicine, Seoul, Korea; 2 Department of Urology, Soonchunhyang University Seoul Hospital, Soonchunhyang University College of Medicine, Seoul, Korea; 3 Department of Urology, Incheon Red Cross Hospital, Incheon, Korea; 4 Department of Urology, Severance Check-Up, Yonsei University Health System, Seoul, Korea; 5 Department of Urology, Gangnam Severance Hospital, Urological Science Institute, Yonsei University College of Medicine, Seoul, Korea

## Abstract

We investigated whether stone heterogeneity index (SHI), which a proxy of such variations, was defined as the standard deviation of a Hounsfield unit (HU) on non-contrast computed tomography (NCCT), can be a novel predictor for shock-wave lithotripsy (SWL) outcomes in patients with ureteral stones. Medical records were obtained from the consecutive database of 1,519 patients who underwent the first session of SWL for urinary stones between 2005 and 2013. Ultimately, 604 patients with radiopaque ureteral stones were eligible for this study. Stone related variables including stone size, mean stone density (MSD), skin-to-stone distance, and SHI were obtained on NCCT. Patients were classified into the low and high SHI groups using mean SHI and compared. One-session success rate in the high SHI group was better than in the low SHI group (74.3% vs. 63.9%, P = 0.008). Multivariate logistic regression analyses revealed that smaller stone size (OR 0.889, 95% CI: 0.841–0.937, P < 0.001), lower MSD (OR 0.995, 95% CI: 0.994–0.996, P < 0.001), and higher SHI (OR 1.011, 95% CI: 1.008–1.014, P < 0.001) were independent predictors of one-session success. The radiologic heterogeneity of urinary stones or SHI was an independent predictor for SWL success in patients with ureteral calculi and a useful clinical parameter for stone fragility.

Despite the widespread acceptance and high success rate of shock-wave lithotripsy (SWL) for urinary stones, some calculi are found to be partially or completely resistant, requiring ancillary procedures with additional costs^1^. Several stone characteristics including stone size, mean stone density (MSD), and skin-to-stone distance (SSD) have been suggested to optimize and predict SWL outcomes.

MSD is well-known as a potentially useful independent predictor of SWL outcomes^2^,^3^,^4^,^5^,^6^,^7^,^8^,^9^,^10^,^11^,^12^,^13^. MSD is the mean value of the Hounsfield units (HUs) of each pixel in a certain stone area that can be easily determined from non-contrast computed tomography (NCCT) using a picture archiving and communication system (PACS)^14^. Generally, a PACS can also provide additional pixel statistics such as the minimum, maximum, and standard deviation of HU values. In statistics, the standard deviation is a measure that is used to quantify the amount of variation or dispersion of a set of data values, and a high standard deviation indicates that the data points are spread out over a wider range of values. Similarly, a high standard deviation of HUs may suggest heterogeneity in stone composition.

As shown in Fig. 1, because the composition of urinary stones can vary even though they have a similar MSD, we postulated that a heterogeneous stone may be more fragile than a homogeneous stone. Herein, we defined the stone heterogeneity index (SHI) as the standard deviation of stone density on NCCT, and investigated whether SHI can be a novel predictor for SWL outcomes in patients with ureteral stones.

## Results

### Demographic analysis

The mean age of total patients was 51.92 ± 14.50 years. The distribution of ureteral stone locations was comprised of 492 cases of upper ureter stones (81.5%), 42 cases of mid-ureter stones (7.0%), and 70 cases of lower ureter stones (11.5%). The mean stone size was 9.12 ± 3.89 mm, and the mean MSD and SHI were 710.25 ± 269.65 HU and 229.45 ± 99.62 HU, respectively. The mean SSD was 109.96 ± 19.31 mm. The one-session success and one-session stone-free rates were 68.9% and 66.2%, respectively (Table 1).

### Comparison between the low and high SHI groups

All patients were divided into low and high SHI groups according to the mean SHI. In terms of age, sex, stone location, size, and SSD, there was no significant difference between the two groups. MSD was significantly higher in the high SHI group than in the low SHI group (817.80 ± 247.02 HU vs. 612.23 ± 251.76 HU, P < 0.001). Nevertheless, the one-session success and stone-free rates in the high SHI group were higher than in the low SHI group (74.3% vs. 63.9%, P = 0.008 in one-session success rate; 70.5% vs. 62.3%, P = 0.043 in one-session stone-free rate; Table 1).

### Factors affecting the one-session success and stone-free status

Univariate and multivariate logistic regression models were generated for one-session success and stone-free status. The multivariate model revealed that smaller stone size (OR 0.889, 95% Confidence Interval [CI]: 0.841–0.937, P < 0.001), lower MSD (OR 0.995, 95% CI: 0.994–0.996, P < 0.001), and higher SHI (OR 1.011, 95% CI: 1.008–1.014, P < 0.001) were independent predictors of one-session success. Similarly, stone size (OR 0.886, 95% CI: 0.839–0.933, P < 0.001), MSD (OR 0.996, 95% CI: 0.995–0.997, P < 0.001), and SHI (OR 1.008, 95% CI: 1.005–1.010, P < 0.001) also had an independent impact on one-session stone-free status (Table 2).

### Impact of SHI on SWL outcomes according to stone size and MSD

In patients with a stone size ≥10 mm, the one-session success rate was 50.2%, and SHI was significantly different between those with success and failure (279.92 ± 115.66 HU vs. 204.99 ± 85.85 HU, P < 0.001). However, there was no difference in SHI according to the success or failure in patients with a stone size < 10 mm (Table 3). Meanwhile, one-session success rates were 75.0% in patients with a MSD < 1000 HU versus 38.0% in patients with a MSD ≥ 1000 HU. In patients with a MSD ≥ 1000 HU, SHI was significantly higher in cases with one-session success than in the cases with failure (308.02 ± 91.87 HU vs. 251.48 ± 54.51 HU, P = 0.001; Table 4).

## Discussion

In the current study, we introduced the concept of radiologic heterogeneity of a urinary stone based on a HU measurement in NCCT and demonstrated the clinical significance of SHI in the management of patients with a ureteral stone. To the best of our knowledge, this is the first report dealing with this novel clinical factor, and we revealed that SHI was an independent predictor of SWL outcomes in ureteral stones. In general, urinary stones with a larger stone size (i.e., >10 mm) or a higher MSD (i.e., >1000 HU) have been deemed to be resistant to SWL, and stone size seems to be the most influential factor in predicting SWL outcomes^1^,^15^. Our results are in agreement with prior studies. Nevertheless, in certain stones with a higher SHI, favorable outcomes can be expected even though a stone may possess conventionally unfavorable clinical features, such as a larger stone size or a higher MSD (Tables 3 and 4). We believe that SHI can be a useful clinical parameter for stone fragility and can play a complementary role for such a clinical prediction in addition to stone size and MSD. In addition, SHI can be readily measured using the currently available PACS without additional equipment.

**Figure 1:**
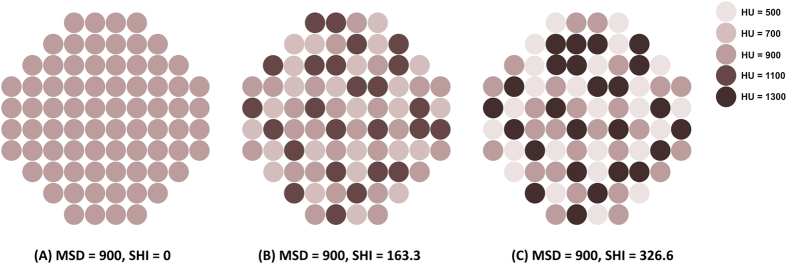
Schematic diagram showing the compositional heterogeneity of urinary calculi. All three stones demonstrate the same mean stone density (MSD) of 900 HU but their stone heterogeneity index (SHI) is different.

MSD has been widely used during the last decade as an important parameter to characterize urinary stones for both research and clinical practice^16^. However, MSD is only an arithmetical average that cannot represent the heterogeneity of stone composition, as shown in Fig. 1. Conversely, the standard deviation of a random variable, statistical population, data set, or probability distribution is the square root of its variance. SHI is an index presenting the radiological heterogeneity of a urinary stone defined as the standard deviation of HU in the region of interest, as aforementioned. Accordingly, SHI can represent the internal diversity of a stone, reflecting not only the heterogeneity of the stone’s composition but also the structural and morphological heterogeneity of a stone. There can be several explanations for the difference in SHI of urinary stones. First, urinary stones are generally not monocrystals, which can be a cause for SHI. Jing *et al.* reported a prospective analysis of urinary calculi composition by infrared spectroscopy with 625 patients in eastern China^17^. They showed that 37.4% of urinary stones are pure stones, but most urinary stones were mixed (62.6%) in which calcium oxalate was the most commonly found major component. Second, the internal structure and morphology of stones can vary, even though the composition of minerals is similar, which can also contribute to such differences in SHI value. Urinary stones present with a variety of gross appearances according to their contour irregularities, such as smoothly round, spiculated, and mulberry stones^18^. Meanwhile, the internal structure showing a heterogeneity of composition or cracks were also detected using NCCT even in stones with the same attenuation; cross-sectional images of such a stone can differ from mottled to lamellar structures^19^. In addition, there can be some empty space within urinary calculi structural irregularities. This space can be filled with water or air, which might be an important cause of heterogeneity of the attenuation index.

Conventionally, stone composition has been undoubtedly important in determining the efficacy of stone treatment, especially SWL. The most dramatic differences have been found with radiolucent uric acid calculi (easily fragmented with SWL) and relatively radiolucent cysteine calculi (often refractory to SWL), which is useful information in selecting stone treatment^20^. Accordingly, knowing the composition of urinary calculi is essential for deciding the optimal mode of treatment. Urine pH, the presence of crystals, urease-positive bacteria in the urine, a plain x-ray, and a history of urinary stones have long been used to predict the composition of stones^21^. During last two decades, the relationship between HU and stone composition has been investigated. Several studies demonstrated with an *in vitro* approach that stone composition could be predicted with high accuracy using HU and HU density (HU divided by the greatest transverse diameter)^22^,^23^,^24^. However, Toricelli *et al.* showed that there was an overlap between the HU values of cysteine and uric acid stones, making it difficult to differentiate these types of stones^25^. Such an overlap of values also precludes any more exact determination of stone composition by the MSD. In 2003, Williams *et al.* suggested that knowing the major composition of a stone may not allow adequate prediction of its fragility in lithotripsy treatment, and variations in internal stone structure, including secondary mineral composition, may be a significant cause of this variability in stone fragility^26^.

The relationship between CT parameters and SWL outcomes has also been extensively investigated, and representative studies are summarized in Table 5. In the NCCT era of urinary stone management, lots of interest has been raised about MSD and SSD as novel predictors of SWL outcomes. Most studies showed that MSD was significantly associated with SWL success, but only two studies showed no relationship between MSD and SWL outcomes^27^,^28^. El-Nahas showed that a MSD > 1000 HU was a significant independent predictor of SWL failure. Thus, they maintained that an alternative treatment should be offered for patients with a MSD > 1000 HU^5^. Interestingly, in cases with a MSD > 1000 HU, the success or stone-free groups demonstrated significantly higher SHIs than the failure group in our study (Table 4). However, the role of SSD as a predictor of SWL success remains controversial. Approximately half of the published studies have advocated the role of SSD in predicting SWL outcomes, but the other studies have failed to demonstrate a significant relationship between SSD and SWL success.

This study has some inherent limitations due to its retrospective design, which may have introduced sampling bias; however, we built a relatively large cohort for ureteral stone disease. In addition, to overcome this type of limitation, subjects of our study were only ureters stones to elucidate the impact of SHI on SWL outcomes more clearly. In renal stone, anatomical considerations including location of calyx and renal pelvic stone or infundibulopelvic angle can be another bias. Two different generating machines may be a bias, but there were no statistical difference in each period. Another concern is that the clinical significance of SHI may be limited due to its OR from the logistic regression analysis, which may be a major obstacle to using SHI in a clinical setting. However, multivariate analysis demonstrated that the predictive power for treatment outcomes was in order of stone size, SHI, and MSD based on odds ratios (Table 2). In addition, SHI was a significant predictor for successful outcomes in patients with stone sizes of ≥10 mm (Table 3), and there was a significant difference in SHI between success and failure groups regardless of MSD (Table 4). Meanwhile, the relationship among stone size, MSD, and SHI is a very important and interesting issue. Correlation analyses demonstrated no relationship between stone size and SHI (r = 0.060; P = 0.114) while showing a positive correlation between stone size and MSD (r = 0.317; P < 0.001). Taken together, although the predictive power of SHI seems to be limited and weaker than stone size, SHI can play a complementary role in the prediction of treatment outcomes, similar to MSD.

However, further studies with a prospective design are needed to confirm our observation on the relationship between SHI and SWL outcomes, and a clinically applicable cut-off value of SHI should be determined for the selection of proper candidates for SWL treatment. In addition, experimental studies in conjunction with chemical and structural analysis of urinary calculi would be helpful for a thorough understanding of the clinical significance of SHI.

In summary, the radiologic heterogeneity of a urinary stone or SHI was independently associated with SWL success in patients with ureteral calculi, thus SHI can be a useful clinical parameter for stone fragility. SHI may be affected by the compositional heterogeneity in urinary calculi, as well as their structural and morphological heterogeneity. SHI will play a promising role when determining a treatment modality in patients with a urinary stone, and especially when selecting the proper SWL candidates from the patients with a stone of large size or high MSD.

## Methods

### Patient cohort

Medical records were obtained from a consecutive database of patients who had undergone the first session of SWL between November 2005 and December 2013 in the Severance Hospital, Seoul, Korea. During this period, a total of 1,519 patients were registered in our database. Inclusion criteria for the current study were a 4- to 20-mm stone in the ureter and radiopaque calculi on a plain x-ray. Patients who did not undergo a NCCT scan were excluded. Ultimately, 604 patients with ureter calculi were eligible for the current analyses. The Institutional Review Board of Severance Hospital approved this study protocol (Approval No. 4-2014-0465).

### Shock-wave lithotripsy

SWL was performed with an electroconductive lithotripter (EDAP Sonolith Praktis, Technomed, Lyon, France) until 2011; after 2012, the device was replaced by an electromagnetic generative lithotripter (Dornier Compact Delta II lithotripter, Dornier MedTech GmbH, Wessling, Germany). All patients were treated under fluoroscopic guidance. The number of shock waves per SWL session varied from 2500 to 4000 at a rate of 60 to 90 shock waves per minute with a launch intensity ranging from 16 to 55 MPa.

### Stone characteristics on non-contrast computed tomography

**Figure 2:**
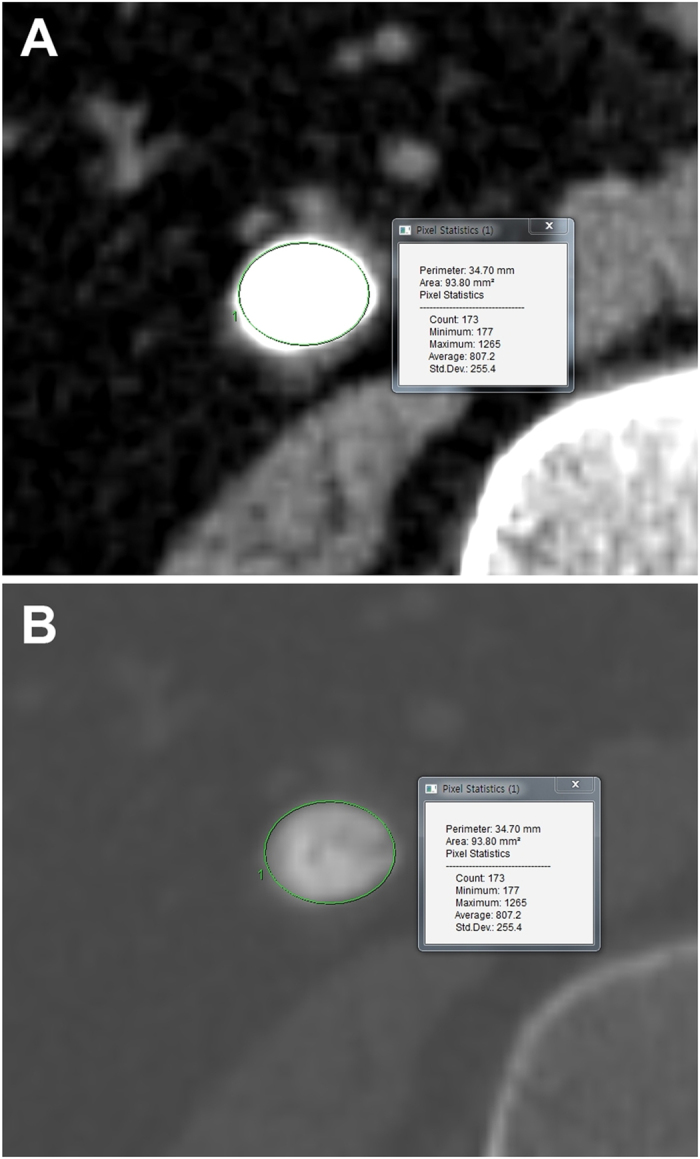
Measurement of stone heterogeneity index (SHI) on axial images of non-contrast computed tomography (NCCT). (**A**) Soft tissue setting view, (**B**) Bone setting view (Mean stone density = 807.2 HU and Stone heterogeneity index = 255.4 HU).

Stone characteristics include the location, size, SSD, MSD, and SHI. We used the GE Centricity system (GE Healthcare Bio-Sciences Corp., Piscataway, NJ, USA) during the measurement procedure. The stone size was determined from the largest stone diameter on the axial or coronal plane of NCCT, and the SSD measurement was taken from the point of the largest stone diameter at a 45° angle from the horizontal. HU was measured on the magnified, axial NCCT image from the point of the largest stone diameter, where the elliptical region of interest incorporated the largest cross-sectional area of the stone without including the adjacent soft tissue. MSD was defined as the mean value of HU in the region of interest, and SHI was defined as the standard deviation of HU in the same region of interest (Fig. 2). A successful SWL treatment of ureteral and renal calculi was defined as those patients who were rendered stone-free or had asymptomatic, clinically insignificant residual fragments ≤3 mm in the largest stone diameter 2 weeks after a single SWL treatment^29^, as measured by a simple x-ray without the need for auxiliary measures within a 3-month follow-up period. Stone-free status was defined as when a simple X-ray analysis determined that patients had a calcification-free 2-week period after a single SWL treatment.

### Statistical analysis

Statistical comparisons of continuous variables from the patient demographic information were carried out using either a Student’s or Welch’s two-sample t-test or the Wilcoxon rank-sum test. Categorical variables were compared using Pearson’s chi-squared test. Univariate and multivariate logistic regressions with a binomial method were carried out for significant factors of one-session success and stone-free status. Statistical analyses were performed using R software (version 3.0.3, R Foundation for Statistical Computing, Vienna, Austria; http://www.r-project.org).

## Additional Information

**How to cite this article**: Lee, J. Y. *et al.* Stone heterogeneity index as the standard deviation of Hounsfield units: A novel predictor for shock-wave lithotripsy outcomes in ureter calculi. *Sci. Rep.* **6**, 23988; doi: 10.1038/srep23988 (2016).

## Figures and Tables

**Table 1:** Clinical data on total patients and patients with low and high stone heterogeneity indices.

	Total patients	Low SHI^a^	High SHI^a^	P-Value
No. of patients	604	316	288	
Sex (M:F)	394:210	204:112	190:98	0.780^b^
Mean Age (yrs)	51.92 ± 14.50	52.17 ± 14.92	51.65 ± 14.05	0.663^c^
Location (%)				0.103^b^
Upper	492 (81.5)	250 (79.1)	242 (84.0)	–
Mid	42 (7.0)	21 (6.6)	21 (7.3)	–
Lower	70 (11.5)	45 (14.3)	25 (8.7)	–
Stone size (mm)	9.12 ± 3.89	9.04 ± 4.56	9.21 ± 3.00	0.567^c^
SSD (mm)	109.96 ± 19.31	110.10 ± 19.94	109.80 ± 18.63	0.850^c^
MSD (HU)	710.25 ± 269.65	612.23 ± 251.76	817.80 ± 247.02	<0.001^c^
SHI (HU)	229.45 ± 99.62	155.50 ± 51.95	310.58 ± 72.54	<0.001^c^
One-session success (%)	416 (68.9)	202 (63.9)	214 (74.3)	0.008^b^
One-session stone-free (%)	400 (66.2)	197 (62.3)	203 (70.5)	0.043^b^

SSD: skin-to-stone distance, MSD: mean stone density, SHI: stone heterogeneity index.

^a^Low and high SHI groups were divided using mean SHI (229.45).

^b^Pearson’s Chi-squared test with Yates’ continuity correction.

^c^Student’s or Welch’s two-sample t-test.

**Table 2:** Univariate and multivariate logistic regression models^a^ on one-session success and stone-free status in total patients with a ureteral stone.

	Univariate			Multivariate		
	OR	95% CI	P-value	OR	95% CI	P-value
One-session success						
Age (year)	0.995	0.983–1.007	0.394			
Sex (Male)	0.831	0.574–1.195	0.323			
Stone size (mm)	0.832	0.789–0.874	<0.001	0.889	0.841–0.937	<0.001
SSD (mm)	0.999	0.990–1.008	0.769			
MSD (HU)	0.997	0.996–0.998	<0.001	0.995	0.994–0.996	<0.001
SHI (HU)	1.004	1.002–1.005	<0.001	1.011	1.008–1.014	<0.001
One–session stone-free						
Age (year)	0.991	0.979–1.003	0.128			
Sex (Male)	0.822	0.573–1.174	0.285			
Stone size (mm)	0.832	0.790–0.875	<0.001	0.886	0.839–0.933	<0.001
SSD (mm)	1.000	0.991–1.008	0.992			
MSD (HU)	0.997	0.996–0.998	<0.001	0.996	0.995–0.997	<0.001
SHI (HU)	1.002	1.000–1.004	0.017	1.008	1.005–1.010	<0.001

SSD: skin-to-stone distance, MSD: mean stone density, SHI: stone heterogeneity index.

^a^A multivariate logistic regression model with forward stepwise selection was performed.

**Table 3:** Comparison of stone heterogeneity index (SHI) in one-session success and stone-free status according to stone size.

		N (%)	SHI (HU)	P-value*
Stone size ≥10 mm (N = 229)				
One-session success	Yes	115 (50.2)	279.92 ± 115.66	<0.001
	No	114 (49.8)	204.99 ± 85.85	
One-session stone-free	Yes	109 (47.6)	274.62 ± 113.76	<0.001
	No	120 (52.4)	213.54 ± 94.76	
Stone size <10 mm (N = 375)				
One-session success	Yes	301 (80.3)	224.15 ± 102.60	0.180
	No	74 (19.7)	210.23 ± 78.89	
One-session stone-free	Yes	291 (77.6)	221.43 ± 100.22	0.991
	No	84 (22.4)	221.31 ± 84.30	

SHI: stone heterogeneity index.

*Student’s two-sample t-test for stone heterogeneity index in each group.

**Table 4:** Comparison of stone heterogeneity index (SHI) in one-session success and stone-free status according to mean stone density.

		N (%)	SHI (HU)	P-value*
MSD ≥ 1000 HU (N = 100)				
One-session success	Yes	38 (38.0)	308.02 ± 91.87	0.001
	No	62 (62.0)	251.48 ± 54.51	
One-session stone-free	Yes	36 (36.0)	299.45 ± 87.65	0.016
	No	64 (64.0)	258.07 ± 64.30	
MSD < 1000 HU (N = 504)				
One-session success	Yes	378 (75.0)	232.69 ± 109.81	<0.001
	No	126 (25.0)	185.19 ± 95.89	
One-session stone-free	Yes	364 (72.2)	229.64 ± 106.64	0.001
	No	140 (27.8)	197.85 ± 98.55	

MSD: mean stone density, SHI: stone heterogeneity index.

*Student’s two-sample t-test for stone heterogeneity index in each group.

**Table 5:** Review of the literature on the relationship between stone characteristics and shock-wave lithotripsy outcomes.

Reference	Year	Country	Size	Stone location	MSD (HU)	SSD (mm)	Predictors of success treatment	
							MSD	SSD
Pareek *et al.*^27^	2005	USA	64	Lower calyx	660/950^b^	81.2/115.3^b^	No	Yes
Gupta *et al.*^2^	2005	India	112	Renal and proximal ureter	–	–	Yes	–
Yoshida *et al.*^3^	2006	Japan	62	Renal and proximal ureter	562/742^a^	–	Yes	–
Perks *et al.*^4^	2007	Canada	76	Renal and ureter	684/1034^a^	–	Yes	–
El-Nahas *et al.*^5^	2007	Egypt	120	Renal	709/776^a^	91/117^a^	Yes	No
Weld *et al.*^6^	2007	USA	200	Renal	662/728^a^	106/106^a^	Yes	No
Perks *et al.*^7^	2008	Canada	111	Renal	801/1092^a^	100/111^a^	Yes	Yes
Kacker *et al.*^8^	2008	USA	325	Renal and ureter	522/724	–	Yes	–
Jacobs *et al.*^30^	2008	USA	85	Renal and ureter	692.9/812.4^c^	103.9/101.0^c^	–	No
Bandi *et al.*^9^	2008	USA	94	Renal and ureter	475/544^d^	108.5/102.9^d^	Yes	No
Ng *et al.*^10^	2009	Hong Kong	94	Proximal ureter	534/626^a^	102.3/104.4^a^	Yes	Yes
Patel *et al.*^28^	2009	USA	83	Renal	787.7/803.2^e^	83.3/107.7^e^	No	Yes
Wiesenthal *et al.*^11^	2010	Canada	422	Renal and ureter	767.2	113.6	Yes	Yes
Choi *et al.*^12^	2012	Korea	153	Ureter	756.5/833.8^f^	103.2/101.0^f^	Yes	No
Tanaka *et al.*^13^	2013	Japan	75	Renal and ureter	670.3	104	Yes	No
Present	2014	Korea	604	Ureter	710.3	110	Yes	No

^a^Success group vs. failure group.

^b^Stone-free *vs.* residual stone (p < 0.05 in both MSD and SSD).

^c^Stone-free *vs*. residual stone (p > 0.05 in both MSD and SSD). SSD was measured from the center of the stone to the level of the skin at 30°.

^d^Stone-free *vs.* residual stone (p > 0.05 in both MSD and SSD).

^e^Stone-free *vs.* residual stone (p > 0.05 in MSD and p < 0.05 in SSD).

^f^Stone size ≤10 mm *vs.* >10 mm.

## References

[b1] Bhojani N. & Lingeman J. E. Shockwave lithotripsy-new concepts and optimizing treatment parameters. Urol Clin North Am 40, 59–66 (2013). 23177635 10.1016/j.ucl.2012.09.001

[b2] Gupta N. P. *et al.* Role of computed tomography with no contrast medium enhancement in predicting the outcome of extracorporeal shock wave lithotripsy for urinary calculi. BJU Int 95, 1285–1288 (2005). 15892818 10.1111/j.1464-410X.2005.05520.x

[b3] Yoshida S. *et al.* Role of volume and attenuation value histogram of urinary stone on noncontrast helical computed tomography as predictor of fragility by extracorporeal shock wave lithotripsy. Urology 68, 33–37 (2006). 16806419 10.1016/j.urology.2006.01.052

[b4] Perks A. E., Gotto G. & Teichman J. M. Shock wave lithotripsy correlates with stone density on preoperative computerized tomography. J Urol 178, 912–915 (2007). 17632139 10.1016/j.juro.2007.05.043

[b5] El-Nahas A. R., El-Assmy A. M., Mansour O. & Sheir K. Z. A prospective multivariate analysis of factors predicting stone disintegration by extracorporeal shock wave lithotripsy: the value of high-resolution noncontrast computed tomography. Eur Urol 51, 1688–1693, discussion 1693–1684 (2007). 17161522 10.1016/j.eururo.2006.11.048

[b6] Weld K. J. *et al.* Shock wave lithotripsy success for renal stones based on patient and stone computed tomography characteristics. Urology 70, 1043–1046; discussion 1046–1047 (2007). 18158009 10.1016/j.urology.2007.07.074

[b7] Perks A. E. *et al.* Stone attenuation and skin-to-stone distance on computed tomography predicts for stone fragmentation by shock wave lithotripsy. Urology 72, 765–769 (2008). 18674803 10.1016/j.urology.2008.05.046

[b8] Kacker R. *et al.* Radiographic parameters on noncontrast computerized tomography predictive of shock wave lithotripsy success. J Urol 179, 1866–1871 (2008). 18353389 10.1016/j.juro.2008.01.038

[b9] Bandi G., Meiners R. J., Pickhardt P. J. & Nakada S. Y. Stone measurement by volumetric three-dimensional computed tomography for predicting the outcome after extracorporeal shock wave lithotripsy. BJU Int 103, 524–528 (2009). 19007365 10.1111/j.1464-410X.2008.08069.x

[b10] Ng C. F. *et al.* Development of a scoring system from noncontrast computerized tomography measurements to improve the selection of upper ureteral stone for extracorporeal shock wave lithotripsy. J Urol 181, 1151–1157 (2009). 19152949 10.1016/j.juro.2008.10.161

[b11] Wiesenthal J. D., Ghiculete D., RJ D. A. H. & Pace K. T. Evaluating the importance of mean stone density and skin-to-stone distance in predicting successful shock wave lithotripsy of renal and ureteric calculi. Urol Res 38, 307–313 (2010). 20625891 10.1007/s00240-010-0295-0

[b12] Choi J. W., Song P. H. & Kim H. T. Predictive factors of the outcome of extracorporeal shockwave lithotripsy for ureteral stones. Korean J Urol 53, 424–430 (2012). 22741053 10.4111/kju.2012.53.6.424 PMC3382694

[b13] Tanaka M. *et al.* Stone attenuation value and cross-sectional area on computed tomography predict the success of shock wave lithotripsy. Korean J Urol 54, 454–459 (2013). 23878688 10.4111/kju.2013.54.7.454 PMC3715709

[b14] Lim K. H. *et al.* Can stone density on plain radiography predict the outcome of extracorporeal shockwave lithotripsy for ureteral stones? Korean J Urol 56, 56–62 (2015). 25598937 10.4111/kju.2015.56.1.56 PMC4294856

[b15] Cakiroglu B. *et al.* Are Hounsfield densities of ureteral stones a predictive factor for effectiveness of extracorporeal shock wave lithotripsy? Int J Clin Exp Med 7, 1276–1283 (2014). 24995083 PMC4073744

[b16] Park S. H. *et al.* Pilot study of low-dose nonenhanced computed tomography with iterative reconstruction for diagnosis of urinary stones. Korean J Urol 55, 581–586 (2014). 25237459 10.4111/kju.2014.55.9.581 PMC4165920

[b17] Jing Z. *et al.* Analysis of urinary calculi composition by infrared spectroscopy: a prospective study of 625 patients in eastern China. Urol Res 38, 111–115 (2010). 20157702 10.1007/s00240-010-0253-x

[b18] Dyer R. B., Chen M. Y. & Zagoria R. J. Abnormal calcifications in the urinary tract. Radiographics 18, 1405–1424 (1998). 9821191 10.1148/radiographics.18.6.9821191

[b19] Williams J. C. Jr. *et al.* High resolution detection of internal structure of renal calculi by helical computerized tomography. J Urol 167, 322–326 (2002). 11743350

[b20] Wolf J. S. Jr. Treatment selection and outcomes: ureteral calculi. Urol Clin North Am 34, 421–430 (2007). 17678991 10.1016/j.ucl.2007.04.010

[b21] Gucuk A. & Uyeturk U. Usefulness of hounsfield unit and density in the assessment and treatment of urinary stones. World J Nephrol 3, 282–286 (2014). 25374823 10.5527/wjn.v3.i4.282 PMC4220362

[b22] Mostafavi M. R., Ernst R. D. & Saltzman B. Accurate determination of chemical composition of urinary calculi by spiral computerized tomography. J Urol 159, 673–675 (1998). 9474123

[b23] Motley G. *et al.* Hounsfield unit density in the determination of urinary stone composition. Urology 58, 170–173 (2001). 11489691 10.1016/s0090-4295(01)01115-3

[b24] Patel S. R., Haleblian G., Zabbo A. & Pareek G. Hounsfield units on computed tomography predict calcium stone subtype composition. Urol Int 83, 175–180 (2009). 19752613 10.1159/000230020

[b25] Torricelli F. C. *et al.* Predicting urinary stone composition based on single-energy noncontrast computed tomography: the challenge of cystine. Urology 83, 1258–1263 (2014). 24726314 10.1016/j.urology.2013.12.066

[b26] Williams J. C. Jr. *et al.* Variability of renal stone fragility in shock wave lithotripsy. Urology 61, 1092–1096, discussion 1097 (2003). 12809867 10.1016/s0090-4295(03)00349-2

[b27] Pareek G., Hedican S. P., Lee F. T. Jr. & Nakada S. Y. Shock wave lithotripsy success determined by skin-to-stone distance on computed tomography. Urology 66, 941–944 (2005). 16286099 10.1016/j.urology.2005.05.011

[b28] Patel T., Kozakowski K., Hruby G. & Gupta M. Skin to stone distance is an independent predictor of stone-free status following shockwave lithotripsy. J Endourol 23, 1383–1385 (2009). 19694526 10.1089/end.2009.0394

[b29] Seitz C. *et al.* Rapid extracorporeal shock wave lithotripsy for proximal ureteral calculi in colic versus noncolic patients. Eur Urol 52, 1223–1227 (2007). 17321666 10.1016/j.eururo.2007.02.001

[b30] Jacobs B. L. *et al.* Effect of skin-to-stone distance on shockwave lithotripsy success. J Endourol 22, 1623–1627 (2008). 18721043 10.1089/end.2008.0169

